# First-time patellar dislocation with resultant habitual dislocation two years later, which was not demonstrated on plain X-rays halfway: a case report

**DOI:** 10.1186/1758-2555-2-23

**Published:** 2010-09-14

**Authors:** Satoshi Ohki, Hiroyuki Enomoto, Eiki Nomura, Hidenori Tanikawa, Yasuo Niki, Hideo Matsumoto, Yoshiaki Toyama, Yasunori Suda

**Affiliations:** 1 Department of Orthopaedic Surgery, Keio University, Tokyo, Japan; 2 Department of Orthopaedic Surgery, International Goodwill Hospital, Yokohama, Japan; 3 Institute of Integrated Sports Medicine, Keio University, Tokyo, Japan

## Abstract

We present an instructive case of habitual left patellar dislocation in which the patella had appeared odd due to lateral tilt relative to contralateral side, but had been radiologically confirmed to be on the trochlea at 1 year prior to the referral. An 11-year-old girl presented to our hospital 2 years after the left patella had dislocated with a 'giving way' when cutting to the left. Our physical and radiological examinations confirmed that the left patella was laterally tilted in the patellar groove with the knee in extension but was dislocated in flexion beyond 45°. In spite of these findings, she had been untreated at the previous hospital since all plain X-rays, including a skyline patellar view, had failed to demonstrate the dislocation. Consequently, in addition to reconstruction of medial patellofemoral ligament, she had to undergo a lateral retinacular release, which might have been unnecessary if treated earlier. This case illustrates that first-time patellar dislocation can gradually lead to habitual dislocation subsequently, and that cautious physical examinations in regard to patella tracking are essential since radiological examinations do not always reveal the pathophysiology of patellar instability.

## Background

Acute patellar dislocation can result in anterior knee pain, recurrent dislocation and patellofemoral arthritis, but rarely in habitual dislocation, defined as a dislocation that occurs every time the knee is flexed [1]. In contrast to recurrent dislocation, which occurs as an isolated and intermittent sequela of injury, the transition to a habitual dislocation after an initial dislocation has not yet been clarified. We report a case of habitual patellar dislocation that appeared odd to the patient's family due to lateral tilt compared with contralateral patella, but was left untreated because plain X-rays (including skyline view) did not demonstrate significant patellofemoral malalignment 1 year prior to the referral.

## Case Report

Two years prior to presentation to our hospital, an 11-year-old girl recognized that her left patella was dislocated with a 'giving way' when cutting to the left. She was capable of repositioning it by herself and saw an orthopedic surgeon who did not point out any skeletal abnormalities on plain X-ray. After 1 year had passed, her father noticed that her knee looked odd; however, it was again diagnosed as intact by another surgeon. Since the deformity gradually became apparent, she was referred to our hospital. Although her body height and weight belonged to the lower 10th percentile, she did not have any associated anomalies that present with patellar instability, such as Down syndrome [2] or Kabuki make-up syndrome [3,4]. She also denied any history of injections into the quadriceps muscle.

**Figure 1:**
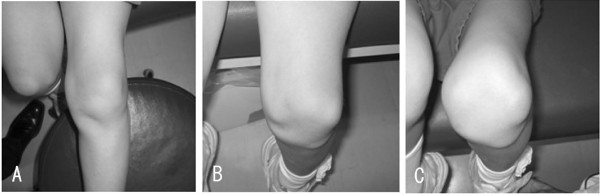
**Left lower extremity Xrays**. Although the patellar dislocation is not apparent with the left knee in extension (Fig. 1-A), it is severely shifted laterally at 90° and 120° of flexion (Fig. 1-B, C),

**Figure 2:**
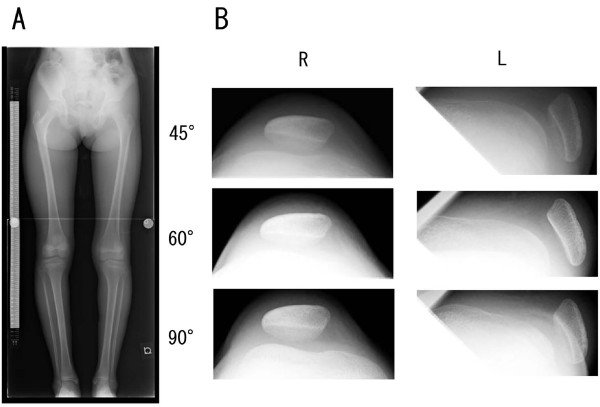
**Bilateral X-rays of the lower extremities, including skyline view**. The modified Q-angle is 36° (Fig. 2-A). The skyline view demonstrates that the patella is dislocated at 45°, 60°, and 90° of flexion in contrast to the contralateral side, which is consistently in position at the femoral trochlea (Fig. 2-B).

**Figure 3:**
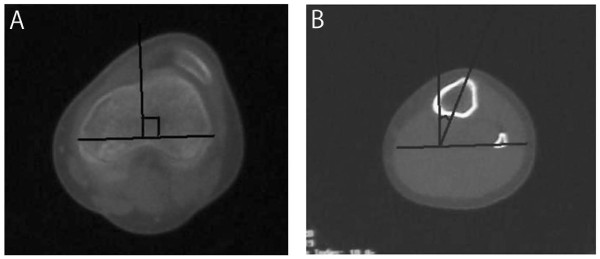
**Measurement of the lateral deviation angle**. The lateral deviation angle, which is defined as the external rotation angle of the tibial tuberosity (Fig. 3-B) relative to the transepicondylar line of the femur (Fig. 3-A), is 36°.

**Figure 4:**
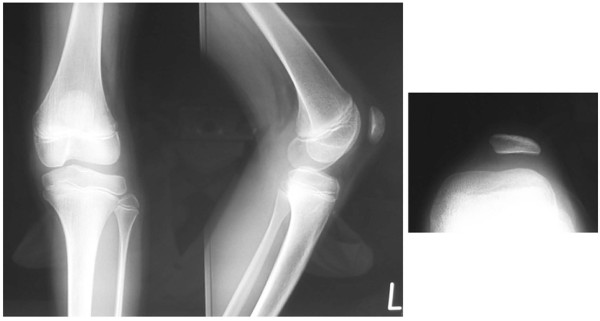
**Plain X-ray taken by the referring physician 1 year after dislocation and 1 year before surgery**. Although the patella locates high (Insall Salvati Ratio; 1.2), and the trochlea is shallow (sulcus angle; 168°), the X-ray confirms that the patella is in position.

Physical examination did not reveal general joint laxity or macroscopically apparent malalignment of the lower extremity. Although the patella was in the femoral trochlear groove in extension, it laterally dislocated with the knee in flexion beyond 45° (Figure 1). She complained slight discomfort by an apprehension test. The passive lateral patellar tilt test was negative since the soft tissue attachment to the lateral border of the patella was diffusely stiff and tight, but no cord-like band was palpable. The patella could be easily displaced laterally due to medial parapatellar instability, but was not so painful even when dislocated. The range of motion at the knee still made it possible for the patient to sit on her legs. On the anteroposterior X-ray of the patient's lower extremities, the modified Q-angle [5] was 36° (normal is between 18.4 and 26°). In contrast to the right patella, which was in normal position, the left patella was laterally dislocated with the knee in flexion at 45, 60, and 90° (Figure 2). The sulcus angle was abnormally high (168°). The lateral deviation angle [5], which indicates the external rotation angle of the tibial tuberosity relative to the transepicondylar line of the femur, was 36° (normal is between 13.1 and 27.3°) when measured by overshadowing of two-level computed tomography images (Figure 3). The primary physician kindly gave us the plain X-ray that was taken at 1 year prior to the referral, which confirmed that the patella was in position with the knee in flexion at 90° (Figure 4). This finding illustrates that the patella had transferred into habitual dislocation during the 1 year prior to the referral.

Since the epiphyseal plates were opened, a proximal realignment by reconstruction of the medial patella femoral ligament (MPFL) combined with a lateral retinaculum release was scheduled. Under anesthesia medial parapatellar instability as well as lateral tightness were significant manually. Intraoperatively, contracture of the lateral patellar retinaculum and scar formation of MPFL were noted. The fibrosis of the vastus lateralis was substantially released to obtain adequate balancing. The MPFL was then reconstructed with Leeds-Keio artificial ligament that was overlapped with the MPFL remnant and medial retinaculum according to previously published articles [6,7]. Using a double-stapling technique, the ligament was fixed to the femoral side just distal to the adductor tubercle while avoiding damage to the growth plate, the color of which could be differentiated from the adjacent bone by macroscopic observation.

**Figure 5:**
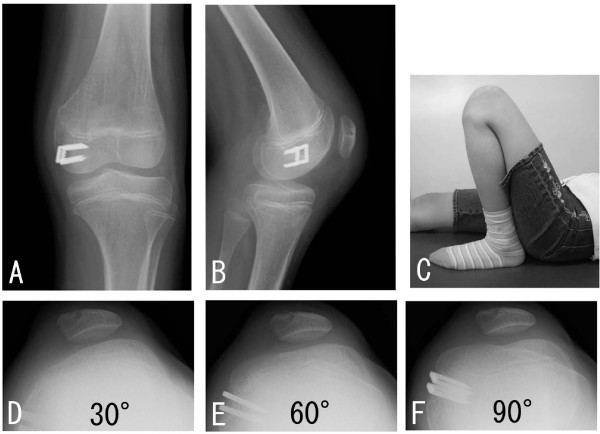
**Images taken during surgery and at 2 years follow-up**. The reconstructed ligament is fixed just distal to the epiphyseal line as well as the adductor tubercle, and just distal to the posterior aspect of the medial epicondyle (Fig. 5-A, B). Two years after the surgery, the ROM is full (Fig. 5-C), and the plain X-ray demonstrates that the patella is consistently in position at 30°, 60°, and 90° in flexion (Fig. 5-D, E, F).

Range of motion exercises were initiated the day after surgery, and partial weight-bearing was allowed using a patellar brace to prevent the patella from being vulnerable to force laterally associated with quadriceps contraction. Two years after the surgery, the patient can extend the knee without lag, and bend her knee the same as preoperatively. The patellar apprehension test was negative, and lateral nor medial parapatellar instability was recognized. The patella was also confirmed to be consistently in position at the femoral trochlea on plain X-ray (Figure 5). The Kujala score improved from 57 to 94.

## Discussion

The clinical significance of this case underscores the difficulties inherent in early diagnosis of habitual patellar dislocation even using the skyline view. Although the patella had looked odd to her father due to tilting compared with contralateral side, it had been radiologically confirmed to be on the trochlea at 1 year prior to the referral. This case also demonstrates the time course from the initial patellar dislocation to habitual dislocation, which has not been reported previously. It implies that first-time patellar dislocation can gradually lead to habitual dislocation as well as recurrent dislocation.

Previous articles have reported that 40 to 60% of patients with first-time patellar dislocation have advanced to recurrent dislocation [8,9] due to several predisposing factors, including patella alta, abnormal patellar morphology, trochlear dysplasia, increased Q angle with lateralized tibial tuberosity, genu valgum, ligament hyperlaxity, external tibial torsion, and increased femoral anteversion [8], [10-13]. In contrast with recurrent dislocation, the factors contributing to the onset of habitual dislocation and its time course from initial dislocation have not been elucidated despite previous reports of an association with quadriceps fibrosis due to muscle injections [14-16], quadriceps contracture [17], and abnormal attachment of the iliotibial tract to the patella [18], none of which were applicable to our case. Among the various parameters evaluated in our case, modified Q angle, sulcus angle, and lateral deviation angle showed aberrations. Further studies, using either meta-analysis or cases series, are needed to determine the factors that predispose an initial dislocation to become a habitual dislocation. Such research will allow more effective treatment of patellar dislocation by predicting the course after first-time dislocation.

In addition to the lateral reticular release, MPFL reconstruction was applied to our case. Since the epiphyseal plate was still open, the femoral attachment site was shifted from the original point to a point just posterior to medial femoral epicondyle and distal to the adductor tubercule and growth plate. Considering the length patterns reported previously [19], it is suggested that the reconstructed ligament be slightly loose in flexion. We have confirmed that the patella in our patient is stable in the femoral groove with the knee in greater than 60° of flexion. At 2 years after surgery, our patient is capable of bending her knee fully without fear of dislocation. Since the patient is in her growth spurt, we will continue close follow-up to ensure a good clinical outcome for our patient.

We emphasize that, had it been suspected 1 year earlier that this case would eventually lead to habitual dislocation, the lateral retinacular release would not have been required [1,20]. Thus, the possibility of habitual dislocation should be considered if patella appears odd and different from the opposite side in extension, even if the radiological examinations do not show apparent dislocation.

## Conclusions

Initial patellar dislocation can gradually lead to resultant habitual dislocation, and cautious physical examinations regarding patella tracking are essential since radiological examinations, including skyline view, do not always reveal the pathophysiology of patellar instability.

## Consent

Written informed consent was obtained from the parent of the patient for publication of this case report and accompanying images. A copy of the written consent is available for review by the Editor-in-Chief of this journal.

## Competing interests

The authors declare that they have no competing interests.

## Authors' contributions

All authors co-wrote the paper and discussed the results and commented on the manuscript. All authors read and approved the final manuscript.

## References

[B1] Shen HC Chao KH Huang GS Pan RY Lee CH Combined proximal and distal realignment procedures to treat the habitual dislocation of the patella in adults Am J Sports Med 2007 35 2101 2108 10.1177/0363546507305014 17724090

[B2] Mendez AA Keret D MacEwen GD Treatment of patellofemoral instability in Down's syndrome Clin Orthop Relat Res 1988 148 158 2970357

[B3] Ikegawa S Sakaguchi R Kimizuka M Yanagisako Y Tokimura F Recurrent dislocation of the patella in Kabuki make-up syndrome J Pediatr Orthop 1993 13 265 267 8459025

[B4] Kurosawa K Kawame H Ochiai Y Nakashima M Tohma T Ohashi H Patellar dislocation in Kabuki syndrome Am J Med Genet 2002 108 160 163 10.1002/ajmg.10247 11857567

[B5] Tsujimoto K Kurosaka M Yoshiya S Mizuno K Radiographic and computed tomographic analysis of the position of the tibial tubercle in recurrent dislocation and subluxation of the patella Am J Knee Surg 2000 13 83 88 11281335

[B6] Nomura E Inoue M Kobayashi S Long-term follow-up and knee osteoarthritis change after medial patellofemoral ligament reconstruction for recurrent patellar dislocation Am J Sports Med 2007 35 1851 1858 10.1177/0363546507306161 17724092

[B7] Nomura E Inoue M Surgical technique and rationale for medial patellofemoral ligament reconstruction for recurrent patellar dislocation Arthroscopy 2003 19 E47 10.1053/jars.2003.50167 12724671

[B8] Larsen E Lauridsen F Conservative treatment of patellar dislocations. Influence of evident factors on the tendency to redislocation and the therapeutic result Clin Orthop Relat Res 1982 131 136 7140059

[B9] Cofield RH Bryan RS Acute dislocation of the patella: results of conservative treatment J Trauma 1977 17 526 531 10.1097/00005373-197707000-00007 875088

[B10] Maenpaa H Lehto MU Patellar dislocation has predisposing factors. A roentgenographic study on lateral and tangential views in patients and healthy controls Knee Surg Sports Traumatol Arthrosc 1996 4 212 216 10.1007/BF01567965 9046505

[B11] Arendt EA Fithian DC Cohen E Current concepts of lateral patella dislocation Clin Sports Med 2002 21 499 519 10.1016/S0278-5919(02)00031-5 12365240

[B12] Atkin DM Fithian DC Marangi KS Stone ML Dobson BE Mendelsohn C Characteristics of patients with primary acute lateral patellar dislocation and their recovery within the first 6 months of injury Am J Sports Med 2000 28 472 479 10921637 10.1177/03635465000280040601

[B13] Sallay PI Poggi J Speer KP Garrett WE Acute dislocation of the patella. A correlative pathoanatomic study Am J Sports Med 1996 24 52 60 10.1177/036354659602400110 8638754

[B14] Bose K Chong KC The clinical manifestations and pathomechanics of contracture of the extensor mechanism of the knee J Bone Joint Surg Br 1976 58-B 478 484 1018035 10.1302/0301-620X.58B4.1018035

[B15] Bergman NR Williams PF Habitual dislocation of the patella in flexion J Bone Joint Surg Br 1988 70 415 419 3372563 10.1302/0301-620X.70B3.3372563

[B16] Lai KA Shen WJ Lin CJ Lin YT Chen CY Chang KC Vastus lateralis fibrosis in habitual patella dislocation: an MRI study in 28 patients Acta Orthop Scand 2000 71 394 398 10.1080/000164700317393402 11028889

[B17] Williams PF Quadriceps contracture J Bone Joint Surg Br 1968 50 278 284 5651335

[B18] Jeffreys TE Recurrent Dislocation of the Patella Due to Abnormal Attachment of the Ilio-Tibial Tract J Bone Joint Surg Br 1963 45 740 743 14074324

[B19] Nomura E Inoue M Hybrid medial patellofemoral ligament reconstruction using the semitendinous tendon for recurrent patellar dislocation: minimum 3 years' follow-up Arthroscopy 2006 22 787 793 10.1016/j.arthro.2006.04.078 16843816

[B20] Colvin AC West RV Patellar instability J Bone Joint Surg Am 2008 90 2751 2762 10.2106/JBJS.H.00211 19047722

